# Modulation of metabolic activity of phagocytes by antihistamines

**DOI:** 10.2478/v10102-011-0004-z

**Published:** 2011-03

**Authors:** Antonin Lojek, Milan Číž, Michaela Pekarová, Gabriela Ambrožová, Ondřej Vašíček, Jana Moravcová, Lukáš Kubala, Katarína Drábiková, Viera Jančinová, Tomáš Perečko, Jana Pečivová, Tatiana Mačičková, Radomír Nosál

**Affiliations:** 1 Institute of Biophysics, Academy of Sciences of the Czech Republic, Královopolská 135, 612 65 Brno, Czech Republic; 2 Institute of Experimental Pharmacology & Toxicology, Slovak Academy of Sciences, Dúbravská cesta 9,841 04 Bratislava, Slovak Republic

**Keywords:** antihistamines, nitric oxide, oxidative burst, phagocytes, reactive oxygen species

## Abstract

The purpose of the study was to investigate the effects of H_1_-antihistamines of the 1^st^ generation (antazoline, bromadryl, brompheniramine, dithiaden, cyclizine, chlorcyclizine, chlorpheniramine, clemastine) and the 2^nd^ generation (acrivastine, ketotifen, and loratadine) on the respiratory burst of phagocytes. Reactive oxygen species generation in neutrophils isolated from rat blood was measured using luminol-enhanced chemiluminescence. Changes in nitrite formation and iNOS protein expression by RAW 264.7 macrophages were analysed using Griess reaction and Western blotting. The antioxidative properties of drugs in cell-free systems were detected spectrophotometrically, luminometrically, fluorimetrically, and amperometrically. The majority of the H_1_-antihistamines tested (bromadryl, brompheniramine, chlorcyclizine, chlorpheniramine, clemastine, dithiaden, and ketotifen) exhibited a significant inhibitory effect on the chemiluminescence activity of phagocytes. H_1_-antihistamines did not show significant scavenging properties against superoxide anion and hydroxyl radical, thus this could not contribute to the inhibition of chemiluminescence. H_1_-antihistamines had a different ability to modulate nitric oxide production by LPS-stimulated macrophages. Bromadryl, clemastine, and dithiaden were the most effective since they inhibited iNOS expression, which was followed by a significant reduction in nitrite levels. H_1_-antihistamines had no scavenging activity against nitric oxide. It can be concluded that the effects observed in the H_1_-antihistamines tested are not mediated exclusively via H_1_-receptor pathway or by direct antioxidative properties. Based on our results, antihistamines not interfering with the microbicidal mechanisms of leukocytes (antazoline, acrivastine and cyclizine) could be used preferentially in infections. Other antihistamines should be used, under pathological conditions accompanied by the overproduction of reactive oxygen species.

## Introduction

The biological effects of histamine are mediated via four types of histamine receptors (H_1_–H_4_) expressed on various cell types (Tiligada *et al*., 2009). The binding of histamine to the H_1_ receptor induces progress of the allergic symptom, which is particularly prevented using histamine H_1_-receptor blockers. These drugs, known as H_1_-antihistamines, are clinically used as anti-allergic or anti-emetic drugs (De Vos, 1999; Drábiková *et al*., 2002). Results published by several authors (*e.g.* Church, 2001) have suggested that the chemical structure of some H_1_-antihistamines – namely positively charged lipophilic molecules – allow them to associate with the cell membrane. Moreover, they are able to inhibit the activity of calcium-dependent enzymes and affect calcium mobilization and discharge of intracellular calcium stores, responsible for various inflammatory reactions including histamine secretion, synthesis of eicosanoids or reactive oxygen species (ROS) generation by phagocytes. NADPH oxidase, located in the plasma membrane and in the membrane of specific granules, produces superoxide anion from which other ROS derive, such as hydrogen peroxide, hypochlorite, and hydroxyl radicals. One quick and sensitive method of measuring the generation of these metabolites is chemiluminescence (CL), as described by Pavelková & Kubala (2004).

Besides ROS, the production of reactive nitrogen species belongs to the important microbicidal tools of professional phagocytes, mainly macrophages, during the fight against pathogenic microorganisms. Nitric oxide (NO), which belongs to the group of reactive nitrogen species, is one of the basic biological mediators which play essential bioregulatory roles in a wide range of processes critical to normal functions in the cardiovascular, nervous, and immune systems. In the presence of inducible nitric oxide synthase (iNOS) in response to inflammatory stimuli, *e.g.* Gram-negative bacterial lipopolysaccharide (Moncada & Higgs, 1993), NO is generated by phagocytes in an L-arginine pathway. Detection of iNOS protein expression and nitrite concentrations are reliable methods for verifying the influence of drugs on NO production by cells (Pekarová *et al*., 2009).

Incomplete information exists about the effects of H_1_-antihistamines on the production of ROS and NO by phagocytes. Therefore, the purpose of the present work was to investigate the changes in ROS generation, nitrite formation, and iNOS protein expression by phagocytes. To describe the direct interactions of H_1_-antihistamines with ROS and NO, the scavenging properties of these drugs against ROS and NO in cell-free systems were also determined.

## Materials and Methods

Antihistamines of the 1^st^ generation – antazoline (ANT), bromadryl (BRO), brompheniramine (BPH), dithiaden (DIT), cyclizine (CYC), chlorcyclizine (CHC), chlorpheniramine (CPH), clemastine (CLE), and the 2^nd^ generation – acrivastine (ACR), ketotifen (KET) and loratadine (LOR) – were purchased from European producers. The stock solutions of drugs (3×10^−3^ M) dissolved in distilled water were stored at −20°C. For the experiments, stock solutions were diluted in Dulbecco's Modified Eagle's Medium (DMEM) supplemented with heat-inactivated 10% fetal bovine serum, and the final concentration of 5×10^−5^ M was tested. Lipopolysaccharide (LPS) from *Escherichia coli* serotype 0111:B4 (Sigma-Aldrich, USA) was dissolved in phosphate buffer solution (1 mg/ml) and stored at −20°C. Other chemicals were purchased from local distributors.

### Cells

Neutrophils isolated from peripheral blood of Wistar rats were used for the analyses of antihistamine effects on the production of reactive oxygen species. Conventional three-month-old male rats were kept under standard conditions in plastic cages, receiving a commercial standard diet with water given *ad libitum*. The experimental protocols were reviewed and approved by the Ethical Committee of the Institute of Biophysics AS CR, in accordance with European Community guidelines. The rats were anesthetized by intraperitoneal administration of ketamine/xylazine (20/2 mg per 100 g of body weight). Five ml of heparinized blood (50 IU/mL) was collected from the right ventricle, and the animals were sacrificed. Erythrocytes were removed after 1h sedimentation and leukocytes with plasma were washed twice in phosphate buffer solution (250×g, 7 min) and resuspended in HBSS to a final concentration of 1×10^6^ PMNL/sample. The respiratory burst of isolated leukocytes was measured according to Pavelková & Kubala (2004), using a microplate luminometer LM-01T (Immunotech, Czech Republic).

Murine leukemic macrophage-like RAW 264.7 cells (ATCC, USA) were used for analyses of H_1_-antihistamine effects on the production of reactive nitrogen species. Cells were grown in DMEM supplemented with 10% fetal bovine serum, gentamycin, glucose, and NaHCO_3_ in a CO_2_ incubator (5% CO_2_ and 95% air humidity) at 37°C. Cells were seeded at an initial density of 2.5×10^6^ cells/ml/well in 6-well tissue culture plates and preincubated with H_1_-antihistamine for 60 min. Cells were subsequently stimulated with LPS in the concentration 0.1 µg/ml and incubated for further 24 h. Non-stimulated cells incubated in the absence of H_1_-antihistamines served as negative control. Cells stimulated with LPS and incubated in the absence of H_1_-antihistamines were used as positive control. After 24 h, supernatants were harvested and the nitrite accumulation was determined. The cells were lysed and used for the measurement of ATP content and iNOS protein expression.

### Chemiluminescence (CL) determination of reactive oxygen and nitrogen production

Briefly, the reaction mixture consisted of 1×10^6^ cells/well in HBSS, 1 mM luminol (stock solution of 10 mM luminol in 0.2 M borate buffer), one of the antihistamines, and 62.5 µg/mL opsonized zymosan particles (OZP). The total reaction volume of 250 µL was adjusted with HBSS. The CL emission was followed up for 1h at 37°C. The area under the obtained curve expressed as relative light units (RLU)/60min was recorded, and the data were expressed as percentage of the positive control.

### Activity of myeloperoxidase

The effect of the antihistamines on the activity of myeloperoxidase (MPO) isolated from lysed HL-60 cells was analysed by bromide-dependent chemiluminescence (Číž *et al*., 2007).

### Evaluation of antioxidant capacity

The antioxidant capacity of H_1_-antihistamines was assessed spectrophotometrically (superoxide anion generated by xanthine/xanthine oxidase), luminometrically (hydroxyl radical generated by Fenton chemistry), and fluorimetrically (peroxyl radical generated by thermal decomposition of 2,2-azobis (2-amidinopropane) hydrochloride according to Nosál’ *et al*., 2005 and Číž *et al*., 2010.

### Testing cell viability

The viability of RAW 264.7 cells was tested by the commercial ATP cellular kit (BioThema, Sweden). Cells were incubated according to the experimental procedure, supernatants were removed, and cells were lysed by the Somatic cell ATP releasing reagent (Sigma Aldrich, USA). Then 50 µl of lysate was mixed with 20 µl of ATP reagent containing D-luciferin, luciferase, and stabilizers. Intracellular ATP contents were determined luminometrically using a luminometer Orion II (Berthold Detection Systems GmbH, Germany).

### Determination of nitrite production by cells

Detection of nitrites (NO_2_^−^) accumulated in the cell supernatants was performed using Griess reagent. The volume of 150 µl of the cell supernatant was incubated with 150 µl Griess reagent (Sigma-Aldrich, USA) for 15min in the dark, at room temperature, and the absorbance was measured at 546 nm using a Spectra Rainbow UV/Vis microplate reader (SLT Tecan, Germany). The concentrations of nitrites were derived from regression analysis using serial dilutions of sodium nitrite as a standard. The concentration values of each sample are expressed as percentage of the positive control.

### Determination of iNOS protein expression in cells

Cells were lysed with 1% SDS lysing buffer with the addition of 1% phenylmethanesulphonylfluoride. The protein volume in the cell samples was determined using commercial BCA protein assay (Pierce, USA). The same concentration of proteins (22 µg) was separated by 7.5% SDS-PAGE and then transferred to a polyvinylidene difluoride membrane (Millipore, USA) in a buffer containing Tris-glycine and 20% methanol. Membranes were incubated with 5% fat-free milk in Tris buffer-Tween 20 (TBS-T) at room temperature for 1 h. The protein was labeled using a mouse antibody (1:1000) specific to iNOS (Anti-iNOS/NOS Type II mAb, BD Transduction Laboratories, USA) and a horseradish peroxidase-conjugated goat anti-mouse IgG antibody (1:2000; ECL™ Anti-mouse IgG, Amersham, Biosciences, USA). The membranes were washed three times in TBS-T buffer for 10 min. Subsequently, immunoreactive bands were visualized using ECL detection reagent (Detection reagents kit, Pierce, USA) and exposed to radiographic film (AGFA, Belgium). Relative levels of proteins were quantified by scanning densitometry using the ImageJ™ program, and the optical density for each individual band was expressed as percentage of the positive control.

### Amperometrical detection of NO scavenging

The scavenging properties of H_1_-antihistamines against NO were evaluated in a chemical system amperometrically, using three electrode systems. Aporphyrinic microsensor working electrode, platinum wire counter electrode, and a miniature saturated silver/silver chloride reference electrode were connected to the ISO-NO MARK II potentiostat (WPI, USA). The measurement was performed using distilled water saturated with pure NO gas (according to the WPI manual and Pekarová *et al*., 2009). The injection of 1 µl of the NO-saturated water into the glass vial (final concentration of NO in the vial=595 nM) caused a rapid increase (peak time=15±5 s) with a subsequent gradual decrease of NO-induced signal until it reached the background current. The potential NO scavenger causes a rapid decrease of the NO-induced signal.

The signal was measured for 280 sto obtain kinetic curves. Then the integral areas under the control curve and sample curves were calculated, and the scavenging activity of the drugs was evaluated.

### Statistical evaluation

Data are expressed as the mean±standard error of the mean (SEM) of at least three independent experiments that were run in duplicates. Results were analyzed by Student's two-tailed t-test using Statistica software (StatSoft, USA), and values below 0.05 (*) were considered statistically significant.

## Results

H_1_-antihistamines were evaluated for their effects on cell viability. None of the compounds tested in the concentration used affected cell viability after 24 h coincubation.

**Figure 1:**
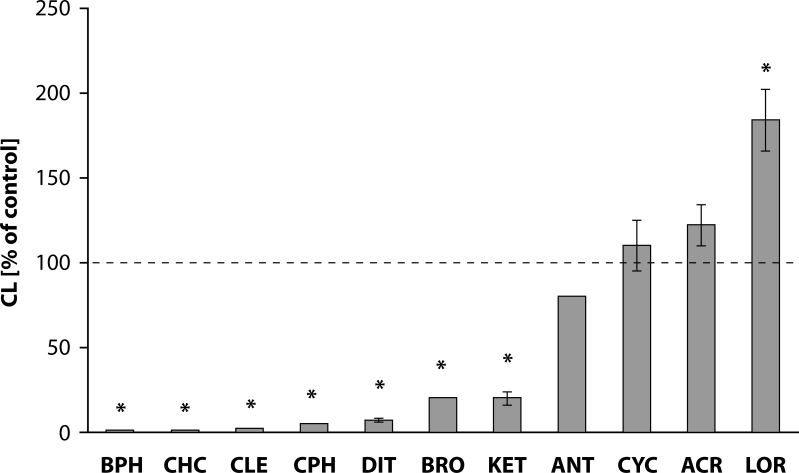
Effects of H_1_-antihistamines in the concentration of 5×10^−5^ M on reactive oxygen species formation in neutrophils isolated from rat blood, measured as their chemiluminescence activity. Data are expressed as mean±SEM of at least three independent experiments, which were run in duplicates. The symbol (*) shows significant differences (*p<*0.05), as compared to control cells.

According to their ability to affect the chemiluminescence response of rat neutrophils isolated from whole blood, H_1_-antihistamines could be divided into three groups (Figure 1). The majority of compounds (bromadryl, brompheniramine, chlorcyclizine, chlorpheniramine, clemastine, dithiaden, and ketotifen) exerted strong, statistically significant inhibition of the chemiluminescence response. On the other hand, loratadine was the only H_1_-antihistamine studied which significantly enhanced the chemiluminescence response of neutrophils. Acrivastine, antazoline, and cyclizine did not exert any effects on the chemiluminescence response of neutrophils.

Since the suppression of neutrophil chemiluminescence could be due to direct scavenging of reactive oxygen metabolites by H_1_-antihistamines, scavenging properties of H_1_-antihistamines against superoxide anion, hydroxyl, and peroxyl radicals were evaluated in a subsequent experiment. None of the H_1_-antihistamines tested influenced myeloperoxidase activity (data not shown). It was found that only bromadryl and dithiaden significantly scavenged peroxyl radicals. Acrivastine and cyclizine scavenged peroxyl radicals insignificantly, and all other H_1_-antihistamines studied had no effect on peroxyl radicals. None of the compounds tested exerted significant effects on superoxide anion or hydroxyl radical generation (data not shown).

Table 1 summarizes the effects of H_1_-antihistamines on NO production by murine RAW264.7 macrophage-like cells. Bromadryl, chlorcyclizine, clemastine, dithiaden, and loratadine significantly suppressed nitrite accumulation, an indirect marker of NO production, in cell supernatants. Some of these compounds (bromadryl, clemastine, dithiaden) and some other H_1_-antihistamines (brompheniramine, ketotifen) significantly inhibited iNOS expression in cells. None of the H_1_-antihistamines tested proved to directly scavenge NO (data not shown).

**Table 1:** Effects of H_1_-antihistamines in the concentration of 5x10^−5^ M on nitrite concentrations in cell supernatants and iNOS protein expression in RAW 264.7 cells stimulated by 0.1 µg/ml LPS.

	NO_2_^−^ concentration	iNOS expression
	
H1-antihistamine	[% of control]	
**BPH**	89.0±4.9	61.0±6.4 *
**CHC**	51.5±0.3 *	86.2±3.0
**CLE**	56.9±4.6 *	40.7±3.7 *
**CPH**	87.3±1.2	81.0±3.7
**DIT**	56.1±3.3 *	50.9±4.0 *
**BRO**	72.7±6.3 *	49.5±4.2 *
**KET**	95.9±0.5	76.5±2.3 *
**ANT**	97.2±1.0	84.3±2.6
**CYC**	88.8±0.8	84.4±4.4
**ACR**	89.6±0.6	98.4±2.1
**LOR**	54.0±1.7 *	84.8±6.4

Data are expressed as mean±SEM of at least three independent experiments, which were run in duplicates. The symbol (*) shows significant differences (*p<*0.05), as compared to control cells.

## Discussion

H_1_-antihistamines play a very important role in the regulation of the metabolic activity of phagocytes. We studied H_1_-antihistamine effects on the production of ROS and NO in isolated rat neutrophils and murine macrophage RAW 264.7 cell line, which is a convenient cell model for monitoring NO production after stimulation with LPS (Yang *et al*., 2009). Cell-free systems were used for the analyses of the antioxidative properties of H_1_-antihistamines (Nosál’ *et al*., 2005).

The majority of the H_1_-antihistamines tested (bromadryl, brompheniramine, chlorcyclizine, chlorpheniramine, clemastine, dithiaden, and ketotifen) exhibited a significant inhibitory effect on the respiratory burst of phagocytes. CL of phagocytes is considered to be dependent on the myeloperoxidase system. However, the MPO activity was not significantly suppressed by H_1_-antihistamines in the concentration tested, thus this effect may be excluded. Direct scavenging of ROS by H_1_-antihistamines could be another possible reason for the decreased CL signal. However, the generation of peroxyl radicals by thermal decomposition of 2,2-azobis (2-amidinopropane) hydrochloride was significantly inhibited only with bromadryl and dithiaden. These H_1_-antihistamines could play a role in the prevention of injury induced by lipid peroxidation. The other antihistamines had no effect in this system. None of the antihistamines showed direct scavenging properties against superoxide anion and hydroxyl radical; thus this could not contribute to the inhibition of CL. Other possible mechanisms for this inhibitory effect by antihistamines, such as interference with calcium ion movement, enzymatic pathways, or second messengers, should be studied (Leurs *et al*., 1995; Church, 2001).

The H_1_-antihistamines tested had different abilities to modulate NO production by LPS-stimulated macrophages. Some of the drugs – including bromadryl, clemastine, and dithiaden – inhibited iNOS expression in LPS-stimulated cells. This inhibition was followed by a significant reduction in nitrite levels. In the presence of brompheniramine and ketotifen, the LPS-induced iNOS protein expression was also inhibited; however, the decrease in nitrite accumulation was less pronounced. On the other hand, chlorcyclizine and loratadine did not affect iNOS protein expression, but they were able to decrease nitrite levels in the cell supernatants. Finally, acrivastine, antazoline, chlorpheniramine, and cyclizine had no effect on nitrite accumulation and iNOS protein expression.

The drugs studied had no scavenging activity against NO in the chemical system. Therefore, we suppose that the inhibitory effect on macrophage iNOS protein expression could be explained by the ability to affect the intracellular signaling pathways that lead to NO production. It was previously documented that the activation of histamine receptors leads to the activation of nuclear factor-κB, which is responsible for the regulation of iNOS expression (Bakker *et al*., 2001). Moreover, the effect of H_1_-antihistamines may be mediated *via* affection of iNOS enzyme activity in cases where the decrease in nitrite accumulation was not accompanied by inhibition of iNOS protein expression.

Our previous results (Králová *et al*., 2009) showed that the structural properties of H_1_-antihistamines, particularly lipophilicity, significantly affected the biological activity of macrophages. On the other hand, the effects of the H_1_-antihistamines tested did not appear to be limited by their hydrophobicity, thus pointing to the involvement of the membrane as their site of action.

It can be concluded that the observed effects of the drugs tested are not mediated exclusively via the H_1_-receptor pathway or by direct antioxidative properties. Based on our results, antihistamines not interfering with the microbicidal mechanisms of leukocytes (antazoline, acrivastine, and cyclizine) could be used preferentially in infections. Conversely, antihistamines inhibiting the production of ROS and NO should be used preferentially under pathological conditions accompanied by overproduction of ROS.

## References

[CIT0001] Bakker RA, Schoonus SB, Smit MJ, Timmerman H, Leurs R (2001). Histamine H(1)-receptor activation of nuclear factor-kappa B: roles for G beta gamma- and G alpha(q/11)-subunits in constitutive and agonist-mediated signaling. Mol Pharmacol.

[CIT0002] Church MK (2001). H(1)-antihistamines and inflammation. Clin Exp Allergy.

[CIT0003] Číž M, Čížová H, Denev P, Kratchanova M, Slavov A, Lojek A (2010). Different methods for control and comparison of the antioxidant properties of vegetables. Food Control.

[CIT0004] Číž M, Komrsková D, Prachařová L, Okénková K, Čížová H, Moravcová A, Jančinová V, Petríková M, Lojek A, Nosál R (2007). Serotonin modulates the oxidative burst of human phagocytes via various mechanisms. Platelets.

[CIT0005] De Vos C (1999). H1-receptor antagonists: effects on leukocytes, myth or reality?. Clin Exp Allergy.

[CIT0006] Drábiková K, Nosál R, Jančinová V, Číž M, Lojek A (2002). Reactive oxygen metabolites production is inhibited by histamine and H_1_-antagonist dithiaden in human PMN-leukocyte. Free Radical Res.

[CIT0007] Králová J, Račková L, Pekarová M, Kubala L, Nosál R, Jančinová V, Číž M, Lojek A (2009). The effects of H_1_-antihistamines on the nitric oxide production by RAW 264.7 cells with respect to their lipophilicity. Int Immunopharmacol.

[CIT0008] Leurs R, Smit MJ, Timmerman H (1995). Molecular pharmacological aspects of histamine receptors. Pharmacol Ther.

[CIT0009] Moncada S, Higgs A (1993). The L-arginine-nitric oxide pathway. N Engl J Med.

[CIT0010] Nosál R, Jančinová V, Číž M, Drábiková K, Lojek A, Fábryová V (2005). Inhibition of chemiluminescence by carvedilol in the cell-free system, whole human blood and blood cells. Scand J Clin Lab Invest.

[CIT0011] Pavelková M, Kubala L (2004). Luminol-, isoluminol- and lucigenin-enhanced chemiluminescence of rat blood phagocytes stimulated with different activators. Luminescence.

[CIT0012] Pekarová M, Králová J, Kubala L, Číž M, Lojek A, Gregor Č, Hrbáč J (2009). Continuous electrochemical monitoring of nitric oxide production in murine macrophage cell line RAW 264.7. Anal Bioanal Chem.

[CIT0013] Tiligada E, Zampeli E, Sander K, Stark H (2009). Histamine H3 and H4 receptors as novel drug targets. Expert Opin Investig Drugs.

[CIT0014] Yang EJ, Yim EY, Song G, Kim GO, Hyun CG (2009). Inhibition of nitric oxide production in lipopolysaccharide-activated RAW 264.7 macrophages by Jeju plant extracts. Interdiscip Toxicol.

